# Susceptibility pattern of uropathogens to ciprofloxacin at the Ghana police hospital

**DOI:** 10.11604/pamj.2015.22.87.6037

**Published:** 2015-10-01

**Authors:** Daniel Kwame Afriyie, Martha Gyansa-Lutterodt, Seth Kwabena Amponsah, George Asare, Vanessa Wiredu, Edem Wormenor, Kwasi Agyei Bugyei

**Affiliations:** 1 Ghana Police Hospital, Pharmacy Department, Accra, Ghana; 2 Department of Pharmacology and Toxicology, University of Ghana School of Pharmacy, Accra, Ghana; 3 Ministry of Health, Accra, Ghana; 4 School of Biomedical and Allied Health Sciences, University of Ghana

**Keywords:** Urinary tract infection, isolates, susceptibility, antibiotics, uropathogens

## Abstract

**Introduction:**

Reports of increasing resistance of uropathogens to antimicrobials is of global concern. Culture and drug susceptibility tests remain a vital guide to effective therapy. The aim of this study was to determine the susceptibility pattern of isolated uropathogens to ciprofloxacin at the Ghana Police Hospital.

**Methods:**

A total of 705 mid-stream urine samples were collected from patients suspected of having urinary tract infection, and visited the Ghana Police Hospital's laboratory from December 2013 to March 2014. Samples were cultured and isolates identified by standard methods, after which isolates susceptibility to ciprofloxacin was determined.

**Results:**

Prevalence of urinary tract infection among patients’ whose samples were analyzed was 15.9%. Predominant uropathogens isolated were *E. coli* (46.4%), *Coliform* (41.1%) and *Coliform* spp. with *Candida* (6.2%). Other isolates were *Pseudomonas* spp. (2.7%), *Salmonella* spp. (1.8%), *Candida* spp. (0.9%) and *Klebsiella spp* (0.9%). The overall resistance among the top three isolated uropathogens to ciprofloxacin was 35.9%. Resistance pattern demonstrated by respective isolates to ciprofloxacin were: *E. coli* (38.5%), *Coliform* (54.3%), and *Coliform* spp. with *Candida* (15%). The other isolates showed 100% sensitivity.

**Conclusion:**

This study revealed a relatively high ciprofloxacin resistance among isolated uropathogens, hence, the need for prudent prescribing and use of ciprofloxacin in urinary tract infection management.

## Introduction

Urinary tract infections (UTIs) are common among inpatients and outpatients, ranking second only to respiratory infections [1]. Worldwide, *Escherichia coli (E. coli)* and *Staphylococcus saprophyticus* are among the predominant isolates of UTIs [2]. Other bacterial isolates found in Sub-Saharan Africa include *Klebsiella pneumonia, Proteus mirabilis, Staphylococcus aureus, Pseudomas aeruginosa* and *Enteroeaseus feacalis* 5. Increasing microbial resistance to conventional antibiotics like beta-lactams, aminoglcosides, sulfonamides and cephalosporins used in UTI management necessitated the use of other agents such as macrolides and fluoroquinolones.

Since their introduction, fluoroquinolones have become a mainstay in the treatment of bacterial infections [6, 7]. Fluoroquinolones are often preferred as first line agents in the management of UTIs, especially when antimicrobial resistance is of concern [8, 9]. They are also recommended where conventional agents are less desirable due to toxicity or hypersensitivity concerns [10]. Ciprofloxacin is the most frequently prescribed fluoroquinolone for UTIs because of its availability in oral and intravenous formulations, favorable bioavailability, and pharmacokinetics which allows twice-daily administration [11, 12]. Resistance to fluoroquinolones has increased markedly since their introduction for treatment of UTI. Rattanaumpawan et *al*, documented fluoroquinolone resistance of 15.8% and 57.4% among *E. coli* and *A. baumannii* respectively [13]. In Africa, substandard drugs, indiscriminate use of antibiotics and erratic prescription by unqualified drug sellers have been identified as contributing to emergence of resistance [14]. Evidence of increasing resistance to ciprofloxacin in some communities is also documented [15, 16]. A recent antimicrobial susceptibility study of isolated uropathogens at the Ghana Police Hospital revealed high resistance to conventional antibiotics [17]. Changes in drug resistance pattern among uropathogens have led to reassessment of local antimicrobial agents [18]. Currently, very little data is available on the susceptibility pattern of uropathogens to ciprofloxacin in Ghana, although the drug is recommended in the national treatment guidelines for UTI management. The aim of this study was to determine the susceptibility pattern of recently isolated uropathogens to ciprofloxacin at the Ghana Police Hospital.

## Methods

### Study population

This was a cross-sectional study that involved analyzing 705 urine samples of suspected UTI patients, who visited the Ghana Police Hospital's laboratory from December 2013 to March 2014. The population consisted of both in- and out-patients.

### Sample collection and processing

Midstream urine samples were collected in sterile urine containers. With a calibrated wire, a loop-full (0.01 ml) of urine was inoculated onto a quarter-plate of Cysteine Lactose Electrolyte-Deficient agar (CLED) (Biotic Laboratories Ltd, U.K) and incubated at 37°C for 24 hours in Nodermann GMBH incubator (Germany). After incubation period, colonies were enumerated and those with significant growth identified. Significant microbial growth was categorized by microbial count greater than 1x10^5^ cfu/ml. Microbial colonies were identified biochemically and serologically according to standard methods [19]. Isolates were speciated by routine procedures which included colonial characteristics, gram-staining, coagulase test and API 20 system. Each colony, representing an isolate was emulsified in 2 ml sterile peptone water, and then transferred into sensitivity agar plates (Biotec laboratories, UK).

### Susceptibility Testing

Susceptibility test was with the Kirby-Bauer disc diffusion method using sensitivity agar (Oxoid, UK) and pertinent antibiotics emphasizing ciprofloxacin with a break point of 5 µg. Inhibition zone diameters pertaining to ciprofloxacin were measured using calipers and compared with standard interpretation charts [20] and scored as sensitive or resistant.

### Data analysis

Data were checked for completeness, entered, and analyzed using SPSS version 20 (Armonk, NY: IBM Corp). Chi-square (χ^2^) was used to test for association and a p value less than 0.05 was considered statistically significant.

### Ethical issues

Protocol for this study was approved by the Ghana Police Hospital Administration.

## Results

Out of the 705 urine samples analyzed, 374 (53%) were obtained from females whilst 331 (47%) were from males. Samples with significant microbial growth (>1x10^5^ cfu/ml) constituted 112 (15.9%), as shown in Table 1. Results also showed a higher incidence (p < 0.0001) of UTI among females 84 (75%) than in male subjects 28 (25%). Predominant uropathogens isolated were *E. coli* 52 (46.4%) and *Coliform* 46 (41.1%). Other isolates found in this study; *Pseudomonas spp., Salmonella spp., Candida spp., Klebsiella*, and *Coliform* spp. with *Candida* are presented in Table 2.

**Table 1:** Level of microbial growth among UTI patients (n = 705)

	Significant growth (>1x10^5^ cfu/ml)	No significant growth
Number of urine cultures	112	593
Percentage of urine cultures	15.9%	84.1%

**Table 2:** Frequency and percent of microbial isolates with significant growth

Isolate	Frequency	Percentage (%)
*E. coli*	52	46.4
*Coliform* spp.	46	41.1
*Coliform* + *Candida* spp.	7	6.2
*Pseudomonas* spp.	3	2.7
*Salmonella* spp.	2	1.8
*Candida* spp.	1	0.9
*Klebsiella* spp.	1	0.9
TOTAL	112	100

Table 3 shows the sensitivity pattern of microbial isolates to ciprofloxacin. *E. coli, Coliform* spp, and mixed *Coliform + Candida* spp. which together constitute 93.7% of total isolates showed sensitivities of 61.5%, 45.7% and 85% to ciprofloxacin respectively. Overall resistance among the top three isolated uropathogens to ciprofloxacin was 35.9%. The rest of the isolates; *Pseudomonas* spp., *Salmonella* spp., *Candida* spp. and *Klebsiella*which constituted 6.3% of total isolates showed 100% sensitivity to ciprofloxacin.

**Table 3:** Sensitivity of isolates to ciprofloxacin

Isolate	Resistant (%)	Sensitive (%)
*E. coli*	38.5	61.5
*Coliform* spp.	54.3	45.7
*Coliform* + *Candida*. spp.	15	85
*Pseudomonas* spp.	—	100
*Salmonella* spp.	—	100
*Candida* spp.	—	100
*Klebsiella* spp.	—	100

## Discussion

This study showed UTI prevalence rate of 15.9%, and its incidence was significantly higher in female than in males. Prevalence rate in this study was lower than findings of 31.6% from a study at the Ghana Police hospital in 2011 [17], and from a similar study in Ghana that reported 56.5% [4]. Multiple predisposing factors have been identified to contribute to higher prevalence among females. A major factor being anatomical differences in the urogenital organs between the two sexes; shorter urethra in females allows quicker access of bacteria to the urinary system [21]. Furthermore, poor personal hygiene coupled with certain cultural practices [22], may account for a higher incidence in women. On the other hand, the antibacterial activity of prostatic fluid in males makes them less susceptible to UTI [23]. Common bacteria isolated from cultures in studies conducted among UTI patients in Ghana include *E. coli, Klebsiella, Candida and Salmonella spp* 25. This study revealed that, *E. coli* and other *coliform* isolates constituted 87.2% of the total microbial isolates. *Pseudomonas spp, Klebsiella spp, Candida spp and Salmonella spp* are others identified among isolates with significant growth. These isolates are similar to those reported in studies done in Ghana [3, 4, 24, 25] and elsewhere [2, 5]. Furthermore, in the current study the two most common isolated uropathogens are the same reported in an earlier study conducted at the Ghana Police Hospital, although their frequencies varied [17]. Also in the previous study,*Staphylococcus aureus and Providencia* were identified among uropathogens.

Incidence of *Candida spp* (6.2%), and mixed *Candida* with *Coliform* (0.9%) in this study was higher than 5.3% reported in a study conducted in Nigeria [26], and above the range (0.14-0.77%) from studies in Israel and Italy [27, 28]. Prevalence of yeast among isolates in the current study was lower than the 11% obtained from a similar study conducted in Kumasi, Ghana [25]. This finding suggests that, clinicians should consider treating possible underlying fungal infections especially when empirical treatment fails in recurrent UTIs. Due to the fact that resistance to antifungals among yeast has been linked to misuse and inappropriate prescription of antifungals [29], medical practitioners should only initiate empirical antifungal treatment when microbial susceptibility tests are unavailable.

Fluoroquinolones are preferred in the treatment of UTI because they have a high bacteriologic and clinical cure rates, as well as low rates of resistance among most common uropathogens [30]. A review of susceptibility trends of uropathogens to ciprofloxacin in the 1990s showed high sensitivities of 93.9% for Enterobacteriaceae, 81.8% for *P. aeruginosa* 90.2% for gram positive aerobes [31], and 80% for gram-negative isolates [32]. The overall sensitivity (64.1%) of the top three isolated uropathogens to ciprofloxacin observed in this study was lower than findings from similar studies [31, 32]. Recently, ciprofloxacin resistant uropathogens have been reported in a number of countries. Those reported include 15% in a study in Turkey [33], 15% in Gaza [12], 14.7% in Spain [15], 33.3% in Nigeria [5], 28.6% in Iran [34], and rates of 46.6 to 59.4% in China [35]. Another study in South Africa revealed ciprofloxacin resistance of 11% for isolates from uncomplicated UTI and 41% for isolates from complicated UTI [36]. Additionally, the overall resistance (35.9%) of the top three uropathogens to ciprofloxacin in this study was relatively high compared with other studies [5, 12, 33, 34]. Although the causative microorganisms of UTI keeps changing over the years, *E. coli* and Enterobacteriaceae remain the most common uropathogens, accounting for about 75-90% of laboratory culture isolates [2]. *E. coli* resistance to ciprofloxacin (38.5%) observed in this study, was lower than the 46.7% observed in a similar study in Ethiopia [37]. However, *E. coli* resistance to ciprofloxacin in this study was higher than similar studies; 32% in Kumasi, Ghana [38], 11% from nine out of ten regions in Ghana [24], 33% in Ethiopia [39], and 22% in Switzerland [40].

In a related study that assessed fecal *E. coli* susceptibility to quinolones between the years 2006 and 2007, it was observed that 13 (52%) of the isolates were resistant to ciprofloxacin, whilst in 2008, 10 (67%) resistant [41]. Although the number of fecal *E. coli*isolates in these studies are fewer than that of isolated from urine, ciprofloxacin resistance among urine *E. coli* appears to be lower than those observed in fecal isolates. Since *E. coli* has been found to be the causative microorganism in over 70% of UTIs worldwide, its resistance to ciprofloxacin observed in this study, and other similar studies in Ghana should be of concern to medical practitioners and drug policy makers. Studies have shown that, pressure on drugs used in therapy can influence resistance pattern of causative organisms than intrinsic bacterial protective mechanisms [42]. In Ghana, the broad therapeutic indications of ciprofloxacin; typhoid fever, infectious diarrhoea, lower respiratory-tract infections, gastro-enteritis, cholera, gonorrhea among others [43], exerts pressure on its use, and increases its risk of misuse. Additionally, although a prescription drug, ciprofloxacin can be obtained over the counter in a number of pharmacies in Ghana, and this further contributes to its misuse.

## Conclusion

The relatively high ciprofloxacin resistance demonstrated by the most predominant isolates, *Coliform* spp. and *E. coli*, in this study should be of great concern to medical practitioners, drug regulatory agencies and policy makers. This emphasizes the need for continuous evaluation of the quality of common antibiotics, prudent drug prescription and judicious use of ciprofloxacin in UTI management.
